# Feasibility and effects of cognitive–motor exergames on fall risk factors in typical and atypical Parkinson’s inpatients: a randomized controlled pilot study

**DOI:** 10.1186/s40001-022-00963-x

**Published:** 2023-01-16

**Authors:** Salome Jäggi, Annina Wachter, Manuela Adcock, Eling D. de Bruin, Jens Carsten Möller, Detlef Marks, Raoul Schweinfurther, Eleftheria Giannouli

**Affiliations:** 1 Department of Health Sciences & Technology, Institute of Human Movement Sciences and Sport, ETH Zurich, Zurich, Switzerland; 2 Division of Physiotherapy, Department of Neurobiology, Care Sciences and Society, Karolinska Institute, Stockholm, Sweden; 3 Department of Health, OST – Eastern Swiss University of Applied Sciences, St.Gallen, Switzerland; 4 Parkinson Center, Center for Neurological Rehabilitation, Zihlschlacht, Switzerland; 5 Department of Neurology, Philipps University, Marburg, Germany; 6 Department of Sport, Exercise and Health, Division of Sports and Exercise Medicine, University of Basel, Basel, Switzerland

**Keywords:** Exergaming, Exercise, Motor-cognitive training, Neurological patients, Parkinson’s disease

## Abstract

**Background:**

People with Parkinson`s disease (PD) often suffer from both motor and cognitive impairments. Simultaneous motor and cognitive training stimulates neurobiological processes which are important especially for people with PD. The aim of this study is to test the feasibility and effects of simultaneous cognitive–motor training in form of exergames in the setting of inpatient rehabilitation of persons with PD.

**Methods:**

Forty participants (72.4 ± 9.54 years; Hoehn and Yahr stage 1–4) were randomly assigned to either the intervention group, which trained five times a week in addition to the conventional rehabilitation program, or the control group, which underwent the standard rehabilitation treatment only. Primary outcome was feasibility (measured by adherence rate, attrition rate, occurrence of adverse events, system usability scale (SUS), and NASA TLX score). In addition, various cognitive (Go/No-Go test, reaction time test (RTT), color word interference test (D-KEFS) and Trail Making Test A and B (TMT)) and motor (preferred gait speed, maximum gait speed, dual-task gait speed, Short Physical Performance Battery (SPPB), Timed Up and Go (TUG) and 5 times Sit-to-Stand (5xStS)) tests were conducted before and after the intervention phase in order to determine training effects

**Results:**

Adherence rate was 97%, there were just two dropouts due to reasons unrelated to the study and there were no adverse events. The mean NASA TLX value was 56.2 and the mean value of the SUS was 76.7. Significant time–group interaction effects were observed for the 5xStS, the SPPB, the RTT, the Go/No-Go test and the D-KEFS 2.

**Discussion:**

Exergaming, as applied in this study, showed to be feasible, safe and likely effective for the improvement of cognitive and motor functions of PD inpatients. Because of this future randomized controlled trials with a main focus on testing the efficacy of this new intervention are warranted.

*Trial registration:* The study has been registered at ClinicalTrials.gov (ID: NCT04872153).

**Supplementary Information:**

The online version contains supplementary material available at 10.1186/s40001-022-00963-x.

## Introduction

Parkinson's disease (PD) is the most common age-related motor neurodegenerative disease worldwide [1]. It causes motor symptoms such as bradykinesia, rigidity, resting tremor and postural instability and is often associated with non-motor symptoms such as cognitive impairment and sleep disorders [2, 3]. Since there is currently no cure for PD, most symptomatic treatments aim to increase brain dopamine levels in order to reduce motor symptoms. There are various pharmacological options (e.g., levodopa) to increase brain dopamine levels and also a surgical option with the implantation of electrodes for deep brain stimulation (DBS). Long-term therapy is difficult to manage, and the medication is often accompanied by side effects and motor fluctuations [4]. The quality of life of people with PD is strongly influenced by the disease consequences, such as an increased fall risk and gait impairment [2, 5].

A high risk of falling and gait impairments can lead to further serious consequences such as reduced physical activity and lower quality of life [6, 7]. Besides pharmacological and surgical treatments, rehabilitation therapies, including physical therapy and exercise therapy, are used to maximize functional abilities and improve quality of life [8]. Physical exercise can improve striatal plasticity and thereby increase the release of dopamine [9]. Various types of exercise training have been used to treat PD over the past few years [9]. The most popular exercise approaches include aerobic exercise, gait training, progressive resistance training, balance training, and complementary exercises. All of these forms of exercise are popular treatments that have positive effects on motor skills, quality of life, and cognition [9]. A new approach to treatment consists of multidisciplinary therapies [10] that should also target cognition [11]. Recent systematic reviews and meta-analyses have shown that motor-cognitive dual-task training has great potential to improve balance, gait and cognition in PD patients and that fall rate can be significantly reduced [12–15].

Exergames combine motor and cognitive training, as cognitive tasks are carried out by executing body movements. Simultaneous motor and cognitive training is believed to stimulate slightly different neurobiological processes, resulting in synergistic response and greater effects on cognitive functioning [16]. In addition, exergaming has positive effects on physical and cognitive skills such as balance, functional mobility, walking ability, and executive functions [17–19]. The gamification of the training increases motivation and training adherence [20–22]. The meta-analysis by Prosperini et al. suggests that exergames are safe for several neurological disorders and improve balance [19]. In addition, initial studies of exergames in inpatient rehabilitation programs in PD patients have shown that exergames are safe, feasible and effective. Adverse effects were rarely reported and were minor [23–26]. However, most of these studies used commercial exergame systems designed for adolescent and recreational purposes rather than for use in rehabilitation. Therefore, an exergame-based training system that has been specially developed for clinical use in older adults could have an even better effect.

The main aim of this study was to test whether an exergame-based cognitive motor training program is feasible within the setting of inpatient rehabilitation, where people with PD have the chance to develop new skills and/or re-learn previous skills with maximum safety due to the intensive supervision of specialists and, thus, would warrant a future full-scale trial. The secondary aim was to get a first impression of the effects of the proposed exergaming intervention on cognitive and physical functions. We hypothesized that exergame training in inpatient rehabilitation for PD patients is feasible and safe and, thus, future trials exploring effectiveness would be warranted. Also, we hypothesized that the effects on cognitive and physical functions in the intervention group are equal to or better than the group receiving conventional rehabilitation therapy only.

## Methods

This paper presents the results of inpatient exergame rehabilitation, which is integrated into the rehabilitation program of people with Parkinson at the Center for Neurological Rehabilitation in Zihlschlacht, Switzerland. This study was part of a series of studies examining the feasibility of exergame training in three different rehabilitation clinics and various inpatient groups [20]. The study was approved by the cantonal ethics committee of Zurich (2020–02388), Switzerland and is registered at ClinicalTrials.gov (ID: NCT04872153). It was conducted in accordance with the Declaration of Helsinki and the Good Clinical Practice (GCP) guidelines. All participants gave written informed consent before participation (Additional file 1).

The reason for conducting this pilot study was scientific, meaning we wanted to determine treatment safety, dose–response effect of the treatment and variance of the treatment effect [27].

### Study design

This pilot study is a randomized controlled trial (RCT) with two arms (one intervention group and one control group) assessing the feasibility of integrating a novel exergame-based cognitive–motor intervention in usual care. Forty patients with an idiopathic or atypical Parkinson’s diagnosis were planned to be recruited for the study. The intervention group received exergame training on the Dividat Senso (Dividat, Schindellegi, Switzerland) 5 times a week in addition to the conventional rehabilitation treatment while the control group received the conventional treatment only. Patients were randomly assigned to one of two study groups using a permuted block randomization with blocks of four using the website randomization.com. Allocation happened directly after the pre assessment by a study member that was not involved in data collection. As a result, the study was only blinded on pre-measurement and randomization. Afterwards blinding was no longer possible because the measurements and interventions were carried out by the same study member. The study was conducted at the Center for Neurological Rehabilitation in Zihlschlacht, Switzerland. The intervention period was adjusted to the length of stay of the individual patients in the clinic and was usually between 2 to 4 weeks. Before and after the intervention, pre- and post-measurements of motor and cognitive functioning were conducted.

Feasibility as primary outcome in this study was adopted as an umbrella term encompassing adherence, attrition, patient acceptability and safety of the intervention.

To assess the effectiveness of the intervention for future full-scale RCTs, secondary outcomes were used to explore the effectiveness. A sample size calculation showed that that in order to correctly reject the null hypothesis with a power of 91%, a total sample size of 16 participants was required. The calculation was based on the effect size (*F* = 0.4) of the interaction effect of the endpoint measure Timed Up and Go (TUG), taken from the study by Morat et al. [28]. Except for the duration of the intervention (more than twice as long) and the conduct of the study with older adults, the exergame intervention was almost identical to the present study. Due to these deviations, which resulted from the significantly shorter intervention period and the more variable population, a sample size of 40 people was aimed in order to also take some drop-outs into account.

### Study participants

The recruitment took place in the rehabilitation clinic Zihlschlacht. All patients were pre-screened for suitability by the patient administration of the clinic. All pre-screened patients were informed about the study verbally and in writing during the first few days after their admission to the clinic. Interested patients were comprehensively informed with a detailed participation information sheet and they had to sign the informed consent. After that, the screening took place, which was carried out by the same study member who then also supervised the measurements and the intervention training. Patients suffering from a Parkinson syndrome were included. The majority of the patients had the diagnosis of PD, but patients with an atypical Parkinson syndrome such as multiple system atrophy (MSA) or progressive supranuclear palsy (PSP) were also allowed to participate. Only patients who fulfilled the following criteria were included: (1) prescription for inpatient rehabilitation; (2) age ≥ 50 years; (3) able to score ≥ 20 at the Mini Mental State Examination (MMSE); (4) able to provide a signed informed consent; (5) physically able to stand for at least 3 min without external support (self-report). Patients with any of the following criteria were excluded: (1) mobility or cognitive limitations or comorbidities which impaired the ability to use the training games and overall system; (2) conservatively treated osteoporotic fractures; (3) previous or current major psychiatric illness (e.g., schizophrenia, bipolar disorder, recurrent major depressive episodes); (4) history of drugs or alcohol abuse; (5) terminal illness; (6) severe sensory impairments (mainly visual, auditory, color blindness); (7) insufficient knowledge of German to understand the training. Then, demographic and medical data were acquired.

### Exergame intervention

Participants of the intervention group conducted cognitive–motor training on the exergaming device Dividat Senso. This training was added to their conventional rehabilitation program. The Dividat Senso is a certified class 1 medical device and has been specifically developed for clinical use in older adults. It consists of a pressure-sensitive platform that records forces generated by movements, such as weight shifts or steps in different directions. The platform includes 20 sensors (strain gauges), 5 vibration motors and an LED control. It is connected to a small computer and a large screen in front of the Senso on which the stimuli of the Dividat exergames are shown. The Dividat exergames target specific cognitive and motor functions that are important for activities of daily living, such as executive and attentional function as well as balance and coordination. The games are played mainly by making steps in four directions (left, right, front, back), but also by shifting body weight.

The training sessions were carried out five times a week. Each training session was planned to last around 15 min, during which participants played five to seven different games. All participants played the same games, with the difficulty level being raised after each week. Furthermore, the training software (DividatPlay) contains an algorithm that adapts the difficulty automatically and in real-time to the performance of a participant. In case of (1) low performance in a game; (2) subjective perception of the patient that the game is too difficult or too easy; (3) and evaluation by the local investigator, the training program could be slightly adjusted by the local study investigator. This ensures personalized training with an adequate training stimulus.

### Control group

Participants of the control group underwent the conventional rehabilitation program and had no training on the Dividat Senso. At the Rehabilitation Clinic Zihlschlacht, the standard procedure of the first week with respect to cognitive–motor therapies consists of 1 × neuro-psychological assessment, 2 × occupational therapy, 2 × fine motor skills therapy, 2 × PD group therapy, 3–4 × individual physiotherapy and daily ergometer cycling. Subsequently, every participant got a program that was individually adjusted to their needs.

### Primary outcomes

The primary outcome of this study was the feasibility of the proposed training in the rehabilitation context for Parkinson patients. Adherence, attrition and the number of adverse events served as feasibility measures. To assess usability and safety, four questionnaires were used, which the participants in the intervention group filled out either after each training (NASA-TLX, enjoyment) or only at post-measurements (SUS, self-created questionnaire with several usability and user experience questions).

### Adherence, attrition and adverse events

Adherence considered the frequency of participants’ attendance at the intervention sessions and attrition considered the proportion of drop-outs. The average adherence rate was calculated as the number of training sessions completed as a percentage of the maximum possible training sessions. Reasons for non-adherence were recorded in the attendance protocol. Adverse events that occurred during the training sessions as well as during the measurements were also recorded in detail by the local study investigator. We a priori adopted a 15% or less attrition rate and 80% or more adherence rate as acceptable for inpatient neurological exergame rehabilitation [29].

### System usability scale (SUS)

The system usability scale (SUS) was used to assess the usability of the Dividat Senso. It is a validated and reliable scale and consists of ten items that are rated on a 5-point Likert scale ranging from 0 to 4 [30, 31].

### Nasa task load index (NASA-TLX) and enjoyment level

The NASA Task Load Index (TLX), developed by Hart and Staveland [32], is a subjective evaluation tool initially developed to assess the workload when working with a human–machine interface system. The questionnaire includes six questions assessing: mental load, physical load, time load, performance, fatigue and frustration. For each question, there is a question that is answered on a 20-point Likert scale from “little” to “too much”. For evaluation, the raw NASA-TLX was used which is an average workload score between 1 and 100 calculated by multiplying each rating by 5. Patients were expected to score an average of 55/100 for the NASA-TLX score [33]. A German translation of the NASA-TLX was used for this study. In addition, the participants were asked after each training session to rate their perceived enjoyment on a 5-point Likert scale.

### Questionnaire regarding usability and safety

Safety was assessed by recording of adverse events and falls that occurred during the intervention. Further, a questionnaire was used to assess user experience and safety aspects. It was only filled in by the intervention group during the post-measurement. The questionnaire included a total of 19 items to evaluate the subjective perception of the exergame training sessions. Thirteen questions (with 1–5 Likert scales) were used to assess following user experience aspects: fun, motivation, excitement and diversification of the games, perceived improvements of motor coordination, perceived improvements of cognitive performance, intention to recommend this type of training for everyone as well as specifically for people with coordinative impairments, or for people with cognitive impairment, frequency of the training sessions, duration of the training sessions, feeling of safety during training, and fear of falling during training. In six further open questions, the participants were asked about their favorite game, their least favorite game, their most challenging game, their least challenging game, their perceived positive effects by the training and general impressions of the training.

### Secondary outcomes

Several physical and cognitive tests that were conducted before and after the intervention or control period (pre- and post-measurements assessing the effects of the exergame intervention on physical and cognitive functions served as secondary outcomes.

### Reaction time test (RTT)

The reaction time test (RTT) is a cognitive–motor test on the Dividat Senso. It measures psychomotor speed based on reaction to visual stimuli. On the screen, six light grey triangles are displayed. Each time one of the triangles went dark, participants had to react as quickly as possible by taking a step in the corresponding direction (front right, front left, right, left, back right and back left).

### Go/no-go test

The Go/No-Go test is another cognitive test performed on the Dividat Senso. This test measures selective attention and inhibition. Participants had to concentrate on a dot in the middle of the screen. Xs (x) and crosses ( +) appeared to the left and right of the dot in random order. Participants were supposed to ignore the crosses and only react to the Xs as quickly as possible by taking a step in the right direction.

### Gait speed

Preferred walking speed in single- and dual-task conditions as well as maximal walking speed in single task-task condition were measured over 7 m on a 11-m track (2 m at the beginning and 2 m at the end of the track served as acceleration/deceleration parts and were not timed). For the dual-task condition, participants had to count backwards in steps of 7 (or in steps of 3 in case serial sevens were impossible) out loud from 250 (at the first trial) or 245 (at the second trial) while walking. They were instructed to start walking and counting backwards as soon as they were given the “Go!” signal. The starting number was given to them right before they started walking. The task was performed twice for each condition and the mean values were used for further analysis. The use of assistive devices was possible and was noted by the local study investigator.

### Trail making test (TMT)

The trail making test is a reliable and valid neuropsychological instrument that measures attention, processing speed and mental flexibility [34–37]. The test consists of two parts. Part A (TMT a) mainly assesses processing speed [36]. Circled numbers [1–25] are randomly allocated on a sheet which the participant had to connect in the correct order. Part B (TMT b) mainly assesses mental flexibility [36, 38, 39]. Participants had to connect the numbers and letters in alternating order. The time required to complete each task was measured, with a maximum duration of 2.5 min in part A and 5 min in part B. If the participant did not finish within this time limit, the last correct letter or the last correct number was noted.

### Color word interference test

The Color Word Interference Test is a version of the Stroop test [40] belonging to the Delis–Kaplan Executive Function System (D-KEFS). This is a reliable and valid test measuring cognitive inhibition and flexibility and consists of four trials [41–43].

In the first trial, a sheet of paper with spots in the colors red, blue and green was shown to the participant. The participant had to name the colors as quickly as possible. In the second trial, the participant had to read out loud written color words as quickly as possible. The words were also “red”, “blue” and “green” printed in black ink. The third trial was the inhibition trial, which is based on the Stroop test [40, 44]. During this trial, the sheet presented to the participant contains the words “red”, “green” and “blue” printed incongruently in red, green or blue ink. The participant was asked to name the ink color of the word as quickly and correctly as possible. In the last condition, the color words were again written in incongruent color. In addition to the third trial, some of the words were framed. The participant had to name the ink color of non-framed words and read the framed words. All of the tasks should be done as quickly and faultlessly as possible. For each trial, time was measured and errors were counted.

### Short physical performance battery (SPPB)

The Short Physical Performance Battery (SPPB) was used as an objective assessment for lower extremity motor functioning in older adults [45]. The test includes three different parts: balance, gait speed and chair stand. In the balance test, participants had to try to keep balance in a side-by-side stand, semi-tandem stand and tandem stand for up to 10 s. Preferred gait speed was calculated from the time participants needed to walk 4 m. For the 5 times Sit-to-Stand test (5xStS), participants had to stand up from a sitting to a standing position as quickly as possible five times in a row. The time taken to complete the task was measured. All subtests (balance, gait speed, chair-stand) were then scored from 0 to 4.

### Timed-up and go (TUG) test

The timed-up and go test requires a chair and a stopwatch. On the start signal, participants had to get up from the chair, walk three meters at a comfortable speed, turn around, walk back and sit down on the chair again. It is a reliable and valid test for quantifying functional mobility and balance, which can be used to detect changes over time [46]. The use of assistive devices was possible and was noted by the local study investigator.

### Statistical analysis

The statistical analysis was conducted with the R Statistics software (RStudio, Inc., Boston, Massachusetts, USA, Version 4.1.1) [47]. First, the data were tested for normal distribution using the Shapiro–Wilk test as well as QQ plots. If the test and the plot suggested that the data were normally distributed, Levene’s test for homogeneity of variance was executed. If normal distribution and equal variances could be assumed, parametric tests were used for analysis. If one of the assumptions was not met, the non-parametric alternative (Friedman’s ANOVA) was used for statistical analysis. Two-way repeated-measures analysis of variance (ANOVA) was used to analyze the differences between pre- and post-measurements in both groups (intervention group and control group). Post hoc tests comparing the different time points were then conducted to enable multiple comparisons. The significance level was set at α  ≤ 0.05 and only data of patients with an adherence ≥ 70% were analyzed (per protocol analysis).

## Results

### Demographics and patient flow

**Fig. 1:**
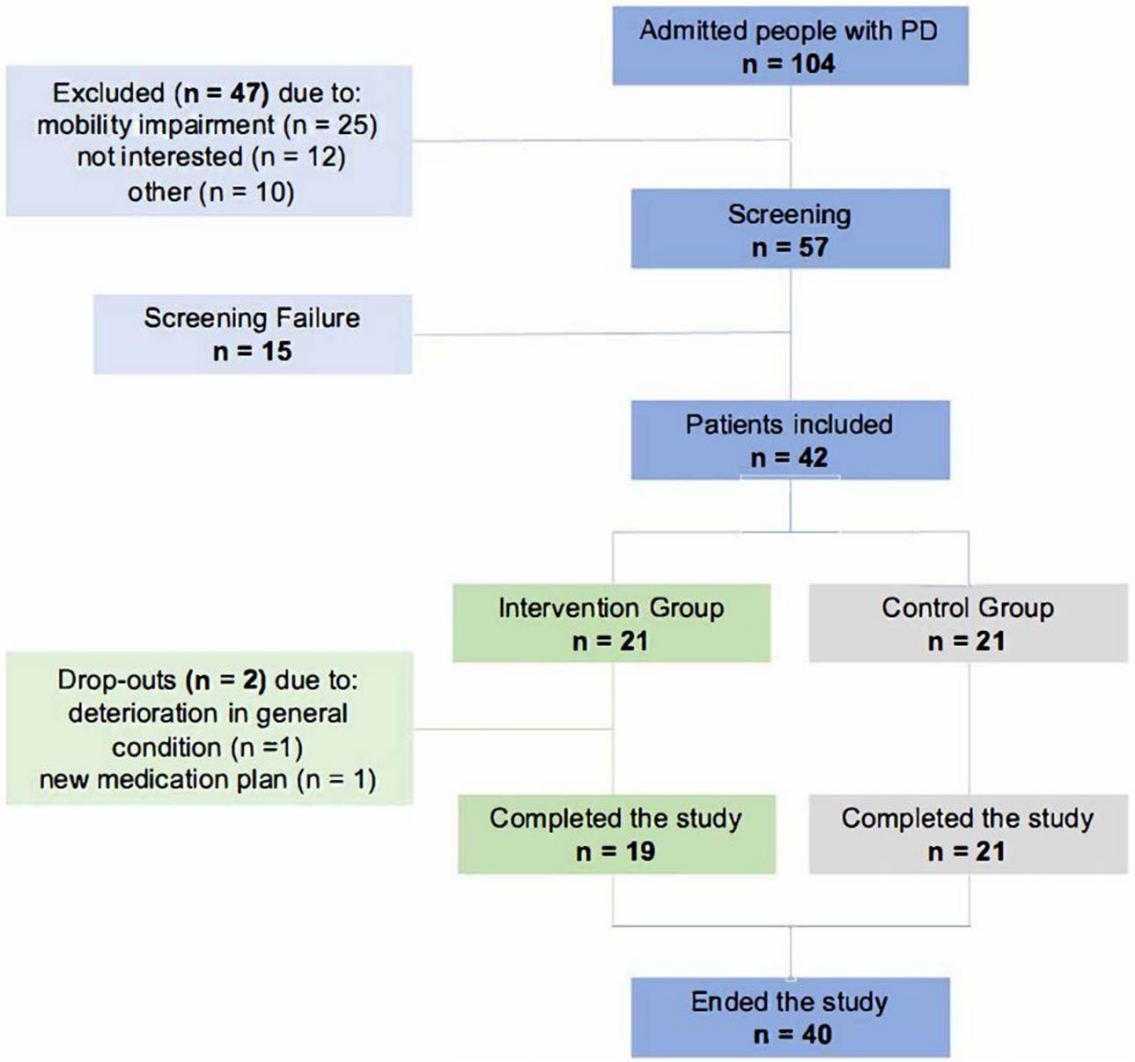
Flow diagram

The demographic data are depicted in Table 1. There was no statistically significant difference between the two groups in terms of age, MMSE score, body mass index (BMI), the time between pre- and post-measurement, years of education or physical activity. Furthermore, the groups did not differ regarding the Levodopa equivalent daily dose and the Movement Disorder Society Unified Parkinson’s Disease Rating Scale (MDS-UPDRS) part II and III at the pre-measurement and the post-measurement (Table 2). A total of 40 patients were included in the final analysis (Fig. 1). The intervention period lasted between 8 and 28 days and the average amount of training sessions was 13.6. Disease duration was significantly different between both groups, which was, however, not reflected by clinical measures of disease severity such as the Hoehn and Yahr stage, the MDS-UPDRS and the Levodopa equivalent daily dose.

**Table 1:** Demographic data

	Intervention group	Control group	*P*-values
Number of participants	19	21	
Sex, [female:male]	[7:12]	[6:15]	
Age, years, mean (SD)	71.89 (9.09)	72.86 (10.14)	0.753
Parkinson’s disease, %	84	95	
MMSE score, mean (SD)	27.79 (1.55)	27.57 (2.71)	0.578
BMI, kg/m^2^	25.8 (3.2)	26.46 (2.55)	0.484
Time between pre- and post-measurement, days, median, (IQR)	22 (9)	21 (4)	0.903
Years of education, years, mean (SD)	13.37 (2.68)	14.24 (3.67)	0.395
Regularly physically active, %	68	48	
Issues with activities of daily living (%)	68	57	
Balance issues (%)	58	52	
Fall within the last 12 months (%)	58	62	
Polypharmacy, %	89	95	
Hoehn and yahr grade (median, IQR)	3 (0.5)	3 (0)	0.896
Diagnoses since, years (mean, SD)	7 (6.68)	12.76 (9.69)	0.034
Dyskinesia (%)	42	38	
Fluctuations (%)	58	48	
Implanted DBS (%)	16	14	

Data presented as mean or median

*SD* standard deviation, *MMSE* mini-mental state examination, *BMI* body mass index, *IQR* interquartile range, *DBS* deep brain stimulation

**Table 2:** Clinical data

	Intervention group	N	Control group	N	*p*-value
Pre-measurement					
MDS-UPDRS II (mean, SD)	17.00 (6.23)	15	15.06 (5.46)	16	0.37
MDS-UPDRS III (mean, SD)	38.50 (13.62)	15	31.53 (11.98)	16	0.146
Levodopa equivalent daily dose, mg (mean, SD)	851.64 (391.54)	19	900.88 (387.41)	21	0.766
Motor fluctuations, on state (%)	95	19	86	21	
Intake of other central nervous medication (%)	47	19	43	21	
Post-measurement					
MDS-UPDRS II (mean, SD)	13.71 (5.27)	15	12.53 (4.81)	16	0.532
MDS-UPDRS III (mean, SD)	28.36 (10.76)	15	24.12 (8.45)	16	0.242
Levodopa equivalent daily dose, mg (mean, SD)	895.32 (474.09)	19	885.98 (399.41)	21	0.947
Motor fluctuations, on state (%)	95	19	86	21	
Intake of other central nervous medication (%)	47	19	57	21	

### Primary outcomes

The recruitment lasted from March to August 2021. Clinicians were willing to recruit patients and patients were willing to be randomized to one of the treatment arms. In the clinical setting of inpatient rehabilitation, the exergame intervention could be carried out as planned.

**Fig. 2:**
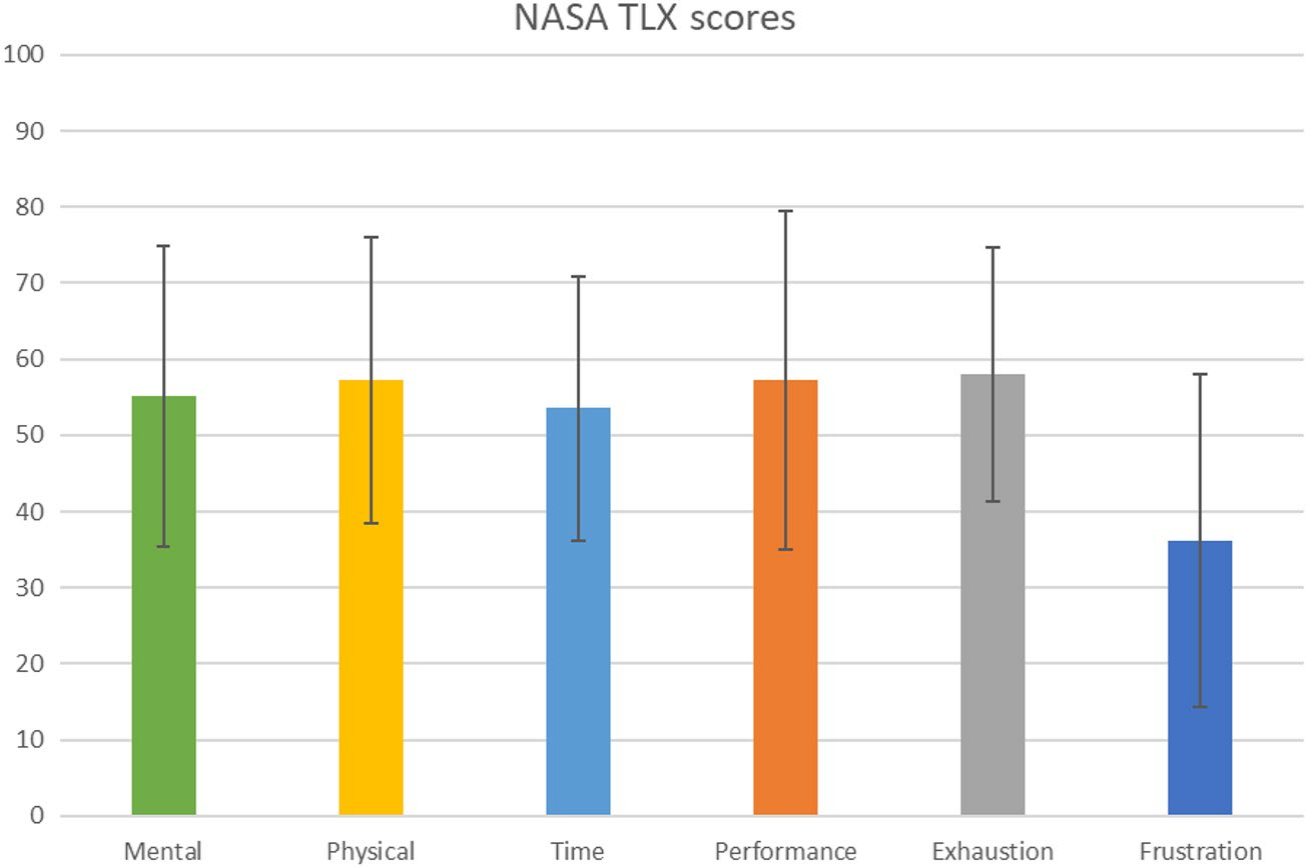
Results of the NASA Task Load Index. The values represent the mean values including the standard deviation of each element of the NASA-TLX, which was required to evaluate the training load

**Fig. 3:**
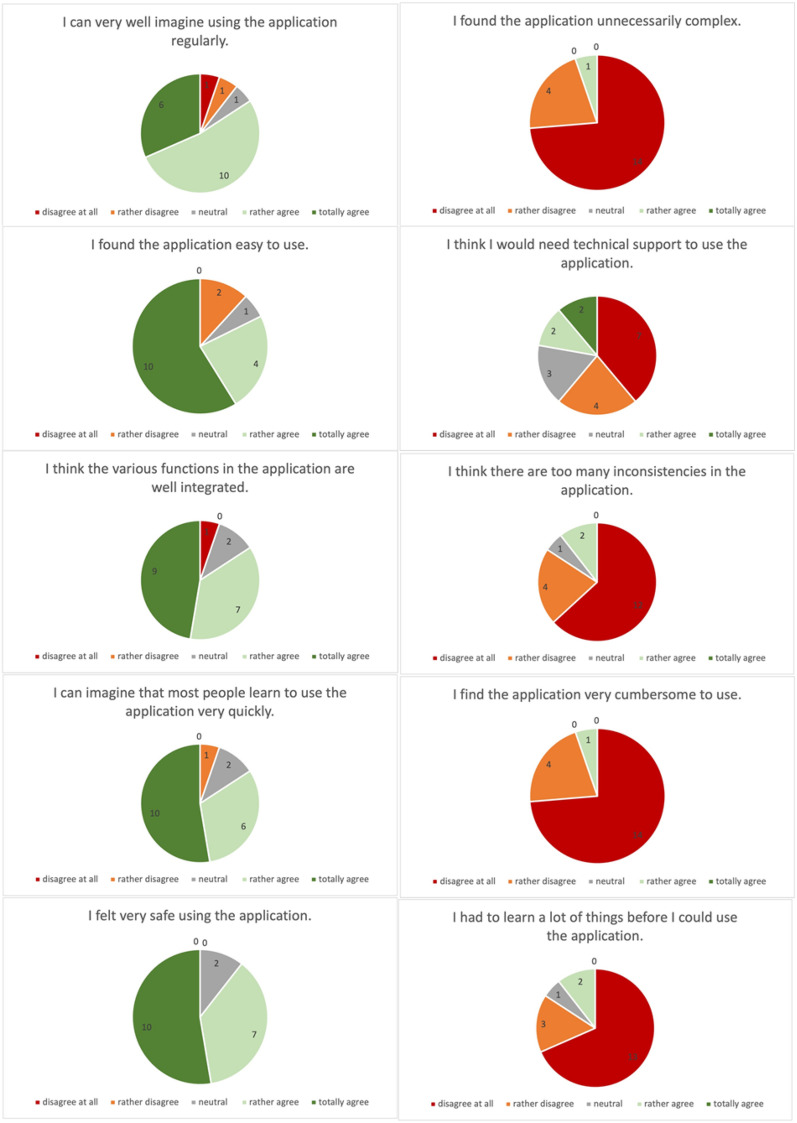
Results of the system usability scale

A total of 104 Parkinson patients were admitted to the clinic over the recruitment period. Some patients were not eligible to participate due to restricted mobility, while others were not interested in participating. Seventy-four percent of the 57 screened participants were finally included and assigned to one of the two groups (Fig. 1). The attrition rate was 5% (*n* = 2 participants) with the reasons for drop-out all unrelated to the study (Fig. 1). One participant had to stop after the first training session because his health deteriorated drastically, and one participant had to stop the study after the 14th training session due to a new therapy and medication plan that was no longer compatible with the study. As a result, there were no drop-outs due to the intervention and, therefore, the intervention-related attrition rate was 0%. All 19 participants in the intervention group who completed the study had an adherence rate of over 70% for the training sessions and were included in the analysis. The overall adherence rate was 96.5%. Reasons for non-adherence were external medical appointments as well as fatigue, acute pain and severe dyskinesia. No adverse events were reported at any time during the study. The mean perceived enjoyment of each training session was 4.51 (SD: 0.73) on a 5-point Likert scale. Mean scores for each item of the raw NASA-TLX are shown in Fig. 2, and the mean total raw NASA-TLX score was 56.19 (SD: 12.49) on a scale of 0–100. The mean SUS score was 76.74 (SD: 6.67) on a scale of 0–100 and the ratings of each SUS item are shown in Fig. 3. The results of the self-questionnaire are summarized in Table 3.

**Table 3:** Self-tailored questionnaire on usability and safety

The training on the dividat senso was fun	Completely true (13)	Quite true (4)	More or less true (2)	Rather untrue (0)	Completely untrue (0)
I was motivated for the training	Completely true (9)	Quite true (9)	More or less true (1)	Rather untrue (0)	Completely untrue (0)
I found the games exciting	Completely true (12)	Quite true (3)	More or less true (4)	Rather untrue (0)	Completely untrue (0)
I found the games diversified	Completely true (14)	Quite true (4)	More or less true (1)	Rather untrue (0)	Completely untrue (0)
I think that the training on the Dividat Senso helped to improve my coordination (e.g., balance, reaction)	Completely true (6)	Quite true (10)	More or less true (3)	Rather untrue (0)	Completely untrue (0)
I think that the Training on the Dividat Senso helped to improve my cognitive functions (e.g., memory, concentration)	Completely true (7)	Quite true (10)	More or less true (1)	Rather untrue (0)	Completely untrue (1)
I would recommend the training on the Dividat Senso to people with coordinative or balance impairments	Completely true (12)	Quite true (4)	More or less true (2)	Rather untrue (0)	Completely untrue (1)
I would recommend the training on the Dividat Senso to people with cognitive impairments	Completely true (9)	Quite true (9)	More or less true (1)	Rather untrue (0)	Completely untrue (0)
I would recommend the training on the Dividat Senso to other people in general	Completely true (11)	Quite true (6)	More or less true (2)	Rather untrue (0)	Completely untrue (0)
How would you rate the frequency of the training sessions (5 × per week)?	Too low (0)	Rather low (2)	Optimal (11)	Rather high (6)	Too high (1)
How would you rate the duration of the training sessions (approx. 10–15 min)?	Too short (1)	Rather short (4)	Optimal (14)	Rather long (0)	Too long (0)
How safe did you feel during the trainings?	Very unsafe (1)	Rather unsafe (3)	Not safe nor unsafe (3)	Rather safe (9)	Very safe (3)
Were you afraid of falling during the training sessions?	Never (16)	Hardly ever (2)	Sometimes (1)	Often (0)	Always (0)
Which game did you like the most?	Targets (8), Habitats (4), Ski (2), Birds (1), Cloudy (1), Flexi (1), Simple (2), other games (0)				
Which game did you like the least?	Hexagon (5), Snake (4), Habitats (3), Tetris (2), Cloudy (1), Divided (1), Flexi (1), Simon (1), Ski (1), other games (0)				
Which game was the most challenging?	Flexi (5), Hexagon (4), Simon (3), Targets (3), Ski (2), Simple (1), Tetris (1), other games (0)				
Which game was the least challenging?	Simple (10), Cloudy (3), Habitats (2), Birds (1), Flexi (1), Simon (1), Snake (1), other games (0)				
Have you noticed any positive effects (physical, psychological, cognitive) during the training period?	Yes (14)	No (5)			
If yes, which positive effects have you noticed?	Reaction (6), balance (5), coordination (5), quickness (5), attention (4), safety (2), motivation to train (1), flexibility (1)				

numbers in brackets represent absolute values of the frequencies in which each answer was given

### Secondary outcomes

**Fig. 4:**
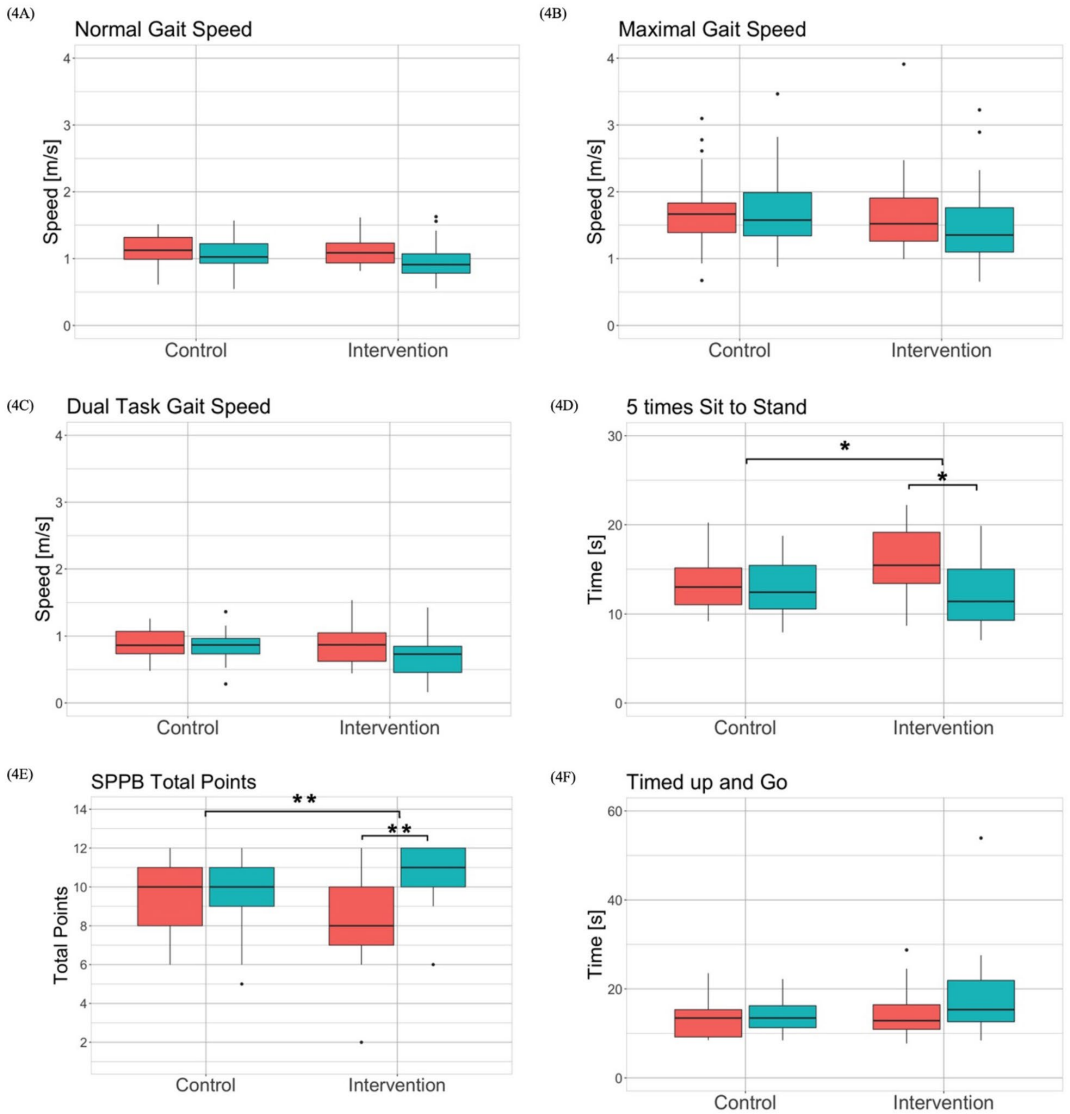
Results of the motor outcomes

**Fig. 5:**
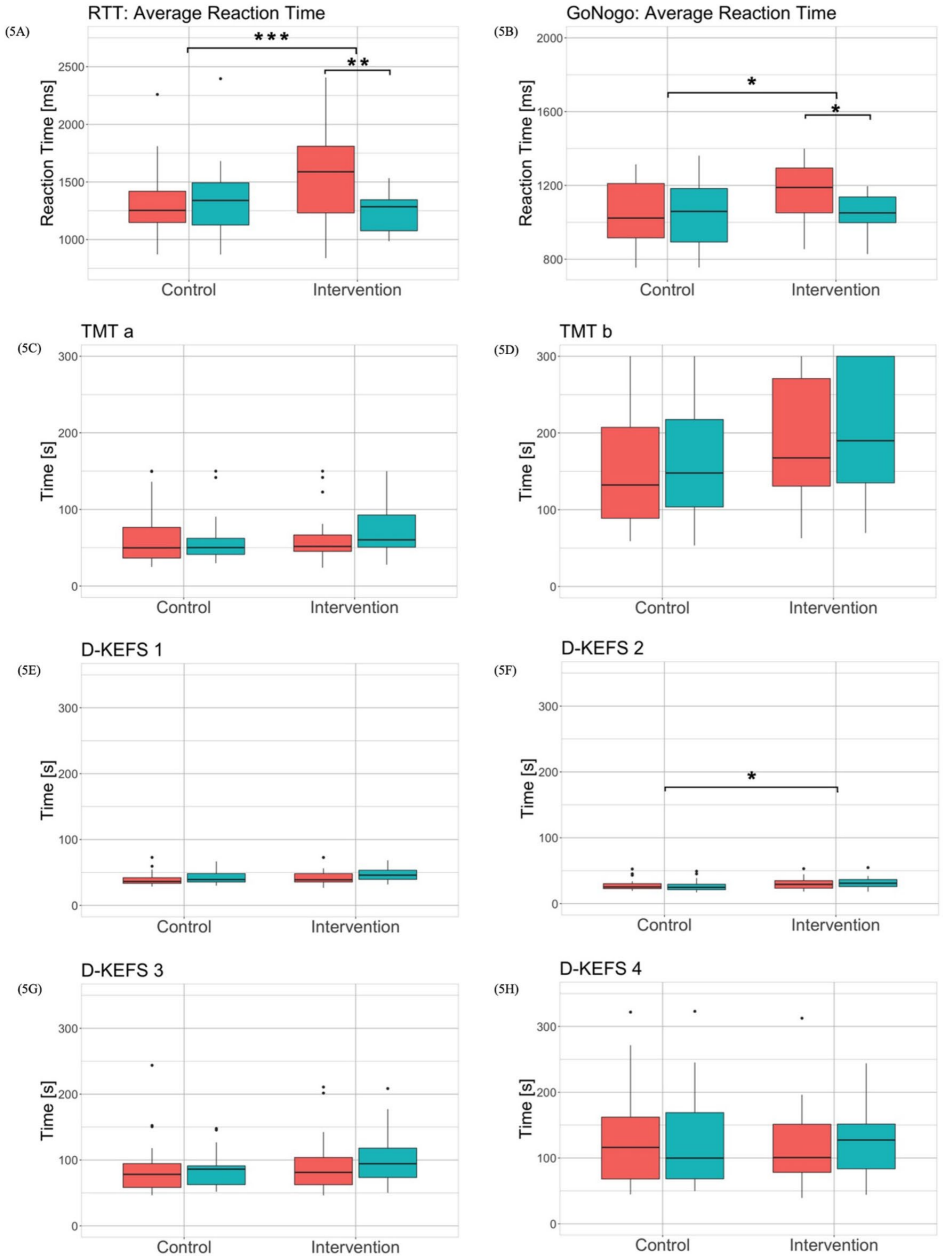
Results of the cognitive outcomes

The Shapiro–Wilk test showed that the data for all outcome measures except normal walking speed, maximal walking speed, dual-task walking speed, 5xStS and Go/No-Go average reaction time were not normally distributed. The Levene test reported non-significant differences between the groups and the time points of each outcome so that the assumption of homogenous variances was fulfilled for all outcomes. The results of the robust two-way mixed ANOVA and the corresponding effect sizes are shown in Table 4. Post hoc tests revealed a significant interaction effect for 5xStS ($\Psi$ = 3.33, *p* = 0.014*), SPPB total score ($\Psi$ = − 2.25, *p* = 0.010*), RTT average reaction time ($\Psi$ = 308.9, *p* < 0.001***), Go/No-Go average reaction time ($\Psi$ = 117.7, *p* = 0.010*) and D-KEFS 2 time ($\Psi$ = − 5.64, *p* < 0.023*). In addition, post hoc tests revealed a significant time effect for normal walking speed ($\Psi$ = − 0.10, *p* < 0.001***), maximal walking speed ($\Psi$ = − 0.14, *p* = 0.023*), dual task walking speed ($\Psi$ = − 0.10, *p* < 0.001***), 5xStS ($\Psi$ = − 2.03, *p* = 0.006**), SPPB total score ($\Psi$ = 1.08, *p* < 0.001***), TUG time ($\Psi$ = 0.92, *p* = 0.012*), Go/No-Go average reaction time ($\Psi$ = − 54.79, *p* = 0.024*),TMT b time ($\Psi$ = 11.43, *p* = 0.028*), D-KEFS 1 time ($\Psi$ = 3.81, *p* < 0.001***) and D-KEFS 3 time ($\Psi$ = 6.28, *p* = 0.020*). Boxplots of the physical and cognitive outcomes are shown in Figs. 4, 5, respectively. The effect sizes allowing to interpret the meaningfulness of change over time in each group are summarized in Table 5.

**Table 4:** Results of outcome measures for intervention and control group at pre- and post-measurement

Outcome measures	Intervention group (IG)		Control group (CG)		Q-value (df), P-value, Effect size (η^2^)		
	Pre	Post	Pre	Post	Pre-Post	IG-CG	Interaction
Normal walking speed (m/s)	0.91 (0.29)	1.09 (0.30)	1.02 (0.29)	1.13 (0.33)	Q(1,22.92) = 20.47 *p* < .001***, *η*^*2*^ = 0.47	Q(1,23.74) = 1.36 *p* = .257, *η*^*2*^ = 0.05	Q(1,22.92) = 3.63 *p* = .069, *η*^*2*^ = 0.14
Maximal walking speed (m/s)	1.35 (0.66)	1.52 (0.65)	1.58 (0.65)	1.67 (0.44)	Q(1,23.76) = 6.70 *p* = .016*, *η*^*2*^ = 0.22	Q(1,22.47) = 0.75 *p* = .397, *η*^*2*^ = 0.03	Q(1,23.76) = 1.84 *p* = .187, *η*^*2*^ = 0.07
Dual-task walking speed (m/s)	0.73 (0.39)	0.87 (0.43)	0.87 (0.23)	0.86 (0.33)	Q(1,24.00) = 9.48 *p* = .005**, *η*^*2*^ = 0.28	Q(1,22.06) = 1.97 *p* = .174, *η*^*2*^ = 0.08	Q(1,24.00) = 4.13 *p* = .053, *η*^*2*^ = 0.15
5xStS (s)	15.45 (5.73)	11.42 (5.73)	13.03 (4.01)	12.45 (4.88)	Q(1,23.94) = 10.44 *p* = .004**, *η*^*2*^ = 0.30	Q(1,21.31) = 0.35 *p *= .563, *η*^*2*^ = 0.02	Q(1,23.94) = 7.79 *p* = .016*, *η*^*2*^ = 0.25
SPPB total score	8.00 (3.00)	11.00 (2.00)	10.00 (3.00)	10.00 (2.00)	Q(1,20.78) = 14.96 *p* < .001***, *η*^*2*^ = 0.42	Q(1,24.00) = 0.21 *p* = .650, *η*^*2*^ = 0.00	Q(1,20.78) = 11.80 *p* = .003**, *η*^*2*^ = 0.36
TUG (s)	15.34 (9.29)	12.85 (5.54)	13.46 (4.95)	13.45 (6.13)	Q(1,20.95) = 8.48 *p* = .008**, *η*^*2*^ = 0.29	Q(1,21.26) = 0.98 *p* = .334, *η*^*2*^ = 0.04	Q(1,20.95) = 4.11 *p* = .056, *η*^*2*^ = 0.16
Go/No-Go average reaction time (ms)	1188.79(242.90)	1051.10 (139.43)	1022.83 (294.54)	1058.07 (290.20)	Q(1,20.36) = 6.39 *p* = .020*, *η*^*2*^ = 0.24	Q(1,20.94) = 1.59 *p* = .222, *η*^*2*^ = 0.07	Q(1,20.36) = 7.58 *p* = .012*, *η*^*2*^ = 0.27
RTT average reaction time (ms)	1587.86 (578.27)	1284.90 (268.76)	1253.46 (271.39)	1339.25 (266.38)	Q(1,19.71) = 9.63 *p* = .006**, *η*^*2*^ = 0.33	Q(1,23.73) = 1.41 *p* = .244, *η*^*2*^ = 0.06	Q(1,19.71) = 17.98 *p* < .001***, *η*^*2*^ = 0.48
TMT a (s)	60.37 (41.92)	51.75 (21.44)	50.27 (21.11)	49.94 (39.99)	Q(1,22.98) = 2.40 *p* = .135, *η*^*2*^ = 0.09	Q(1,23.00) = 1.03 *p* = .321, *η*^*2*^ = 0.04	Q(1,22.98) = 2.40 *p* = .135, *η*^*2*^ = 0.09
TMT b (s)	189.91 (165.04)	167.54 (140.31)	147.87 (113.94)	132.22 (118.47)	Q(1,23.95) = 5.84 ***p*** = .024*, *η*^*2*^ = 0.20	Q(1,21.87) = 1.93 *p* = .178, *η*^*2*^ = 0.08	Q(1,23.95) = 0.03 *p* = .876, *η*^*2*^ = 0.00
D-KEFS 1 time (s)	45.90 (13.84)	38.81 (12.78)	39.16 (12.88)	36.25 (8.72)	Q(1,17.10) = 25.60 *p* < .001***, *η*^*2*^ = 0.60	Q(1,23.26) = 2.06 *p* = .165, *η*^2^ = 0.08	Q(1,17.10) = 2.15 *p* = .161, *η*^*2*^ = 0.11
D-KEFS 2 time (s)	30.94 (10.67)	29.18 (11.43)	24.66 (8.27)	25.25 (7.51)	Q(1,19.41) = 0.12 *p* = .730, *η*^*2*^ = 0.00	Q(1,20.62) = 3.44 *p* = .078, *η*^*2*^ = 0.14	Q(1,19.41) = 6.06 *p* = .023*, *η*^*2*^ = 0.24
D-KEFS 3 time (s)	94.37 (44.60)	81.23 (41.33)	86.16 (28.50)	78.21 (36.16)	Q(1,17.29) = 6.40 *p* = .021*, *η*^*2*^ = 0.27	Q(1,22.32) = 1.10 *p* = .305, *η*^*2*^ = 0.05	Q(1,117.29) = 1.22 *p* = .2848, *η*^*2*^ = 0.07
D-KEFS 4 time (s)	127.33 (68.04)	100.68 (73.13)	99.85 (100.63)	116.13 (94.06)	Q(1,23.98) = 0.40 *p* = .534, *η*^*2*^ = 0.02	Q(1,23.58) = 0.00 *p* = .982, *η*^*2*^ = 0.00	Q(1,23.98) = 1.07 *p* = .311, *η*^*2*^ = 0.04

Number of participants (*n*) = 40, n in *IG* = 19, n in *CG* = 21, data presented as median (IQR = interquartile range), *5xSTS* = 5 times standing up from a chair (part of SPPB)

*SPPB* Short Physical Performance Battery, *TUG* timed up and go, *RTT* reaction time test, *TMT* trail making test, *D-KEFS 1* color naming trial, *D-KEFS 2* word reading trial, *D-KEFS 3* inhibition trial, *D-KEFS 4* inhibition/switching trial

**Table 5:** Effect sizes representing change over time of the intervention and control group

Outcome measures	Intervention group (IG)	Control group (CG)
	Pre–post	Pre–post
Normal walking speed	*r* = 0.71, ***p*** < 0.001***	*r* = 0.39, *p* = 0.076
Maximal walking speed	*r* = 0.59, ***p*** = 0.008**	*r* = 0.12, *p* = 0.609
Dual-task walking speed	*r* = 0.76, ***p*** < 0.001***	*r* = 0.18, *p* = 0.432
5xStS	*r* = 0.76, ***p*** = 0.001**	*r* = 0.24, *p* = 0.312
SPPB total score	*r* = 0.78, ***p*** < 0.001***	*r* = 0.14, *p* = 0.619
TUG	*r* = 0.62, ***p*** = 0.005**	*r* = 0.18, *p* = 0.432
Go/No-Go Average reaction time	*r* = 0.68, ***p*** = 0.001**	*r* = 0.00, *p* = 0.957
RTT average reaction time	*r* = 0.75, ***p*** < 0.001***	*r* = 0.07, *p* = 0.759
TMT a	*r* = 0.48, ***p*** = 0.040*	*r* = 0.12, *p* = 0.609
TMT b	*r* = 0.55, ***p*** = 0.021*	*r *= 0.25, *p* = 0.205
D-KEFS 1 time	*r* = 0.73, ***p*** < 0.001***	*r* = 0.38, *p* = 089
D-KEFS 2 time	*r* = 0.40, *p* = 0.087	*r* = 0.14, *p* = 0.539
D-KEFS 3 time	*r* = 0.48, ***p*** = 0.036*	r = 0.24, *p* = 0.288
D-KEFS 4 time	*r* = 0.36, *P* = 0.130	*r* = 0.05, *p* = 0.838

Number of participants (*n*) = 40, n in *IG* = 19, n in *CG* = 21

## Discussion

### Feasibility outcomes

The main aim of this study was to test the feasibility of integrating an exergame intervention in the neurological inpatient rehabilitation of patients with PD in the sense that we wanted to determine treatment safety, dose–response effect of the treatment and variance of the treatment effect. The main message of our pilot trial is that the process of integrating this novel treatment option into a clinical setting is doable and safe. Furthermore, the chosen dose level of the intervention elicits a positive response in the intervention group patients with promising effect size estimations. As all outcome measures in terms of usability, safety and acceptance indicate a high level of feasibility, a full RCT of the intervention would be warranted. The adherence rate of 97% is very high and is consistent with previous studies that have examined the adherence of technology-based exercise interventions in older adults [20, 48]. As participants were closely monitored throughout the entire training process, therapeutic presence could also have contributed to the high adherence rate [49]. During the study, many participants emphasized that they value one-to-one supervision from the same person. This could also have a positive effect on adherence [50]. A very low attrition rate was also observed and there was no drop-out due to intervention-related reasons. This results in an intervention-related attrition rate of 0%. In addition, both the high adherence and the low attrition rate can be explained by the high motivational potential of exergames [22, 51–54]. The participants in this study were highly motivated and enjoyed exercising on the Dividat Senso, which is also consistent with previous findings that older people with PD like to play exergames [48, 55]. The supervision of the local investigator and the handrail to hold onto, which the Dividat Senso has, reduced the risk of falls and thus eliminated another important factor that discourages people with PD from exercising [56]. This resulted in the training being generally perceived as safe. Very few participants were afraid of falling during the training session. From that, it can be concluded that exergaming on the Dividat Senso is a safe training method for patients with PD.

Looking at the average NASA-TLX raw value of 56.2 points, it is noticeable that this corresponds to the expected value of 55. From this together with the observed interaction effects in favor of the experimental group it can be concluded that the training load was sufficient. As Grier [33] summarizes in a meta-analysis, the value of 56.2 is a little lower but comparable to performing physical activities (mean: 62.0) but massively higher than for cognitive tasks (mean 46.0). This could be because patients with PD generally have more problems with motion and motor control [57]. The illness reduces mental flexibility and adaptability and also slows down the ability to react [2, 3, 5, 58]. As the games on the Dividat Senso require full attention and quick reactions, this can quickly be perceived as more cognitively exhausting by participants with PD than it is for others. The cognitive component was therefore perceived as more difficult, while the physical component of the training was easier compared to other physical activities. Most of the participants rated the training sessions as optimal in terms of duration and frequency. This could mean that the training effort was perceived as pleasant and effective despite the high NASA-TLX. It could also indicate that this type of training intervention is judged by the level of physical effort rather than cognitive effort. With a SUS score of 77, the usability of the Dividat Senso can be rated as good [59]. Only technical support would be required for optimal use of the device, but this can be ensured by the therapist in the clinical area. For the participants, however, the intervention phase and the training with the Dividat Senso were satisfactory and there were even subjectively positive effects, which certainly contributed to the high level of motivation. In summary, this study was able to show for the first time that exergaming on the Dividat Senso is feasible for patients with PD in terms of usability, safety and acceptance. In this way, exergaming can be successfully integrated into the rehabilitation program of patients with PD in inpatient rehabilitation clinics.

### Physical and cognitive function outcomes

The effects of additional cognitive–motor training on specific physical and cognitive functions were examined. In all physical and cognitive outcome measures, the intervention group that received additional exergame training achieved the same or higher performance improvements compared to the control group. Both groups, however, did not improve significantly in most physical and cognitive outcome measures during the intervention period. Only the intervention group improved significantly in the 5 times Sit-to-Stand test (5xStS), the Short Physical Performance Battery (SPPB), in the two cognitive tests on the Dividat Senso RTT and Go/No-Go and in the D-KEFS 2 time test. The effect sizes were between 0.00 and 0.78 and were always in favor of the intervention group (Table 5). A significant interaction effect between group and time showed that group assignment had a significant influence on the performance of 5xStS, RTT, Go/No-Go and D-KEFS 2 time test. In the intervention group, an improvement in choice reaction time, as also demonstrated by Crotty et al. [60], could thus be shown. Further improvements in cognitive tasks were shown by the tests on the Dividat Senso as well as the D-KEFS 2 time test and the 5xStS reported improvement in functional strength of the lower extremities as a motor functions. These significant differences between the groups in terms of outcome measures assessing physical or cognitive function alone have also been reported in previous studies. Effects of exergaming on cognitive function [61], balance and motor function [57, 59] have been reported. However, no significant improvement in walking speed could be determined which contrasts other studies [58]. In those studies, however, the intervention period was longer, usually lasting 8 to 12 weeks. Additionally, instead of the SPPB the Berg Balance Scale (BBS) was used as a measurement tool for balance. Nevertheless, the performance of the motor and cognitive functions of the intervention group were at least equivalent to those of the control group as stated in other studies [14, 22, 62–64]. This study`s results warrant further definitive RCTs.

### Study limitations

The study had some limitations, which must be acknowledged. First, the exergame intervention was investigated in PD patients during inpatient rehabilitation, therefore generalization to other populations and settings is limited. Further studies are required to test the feasibility and effects of exergaming on the Dividat Senso in other patient groups in inpatient rehabilitation, but also in people with PD and other patient groups in outpatient rehabilitation over a longer period of time. Another limitation of this study is the short duration of the intervention period. The standard prescription for a stay in neurological rehabilitation for people with PD in Switzerland is 3 weeks with the possibility to extend another week. In order to determine effectiveness more clearly, this period should be extended. However, as this study aimed to test the feasibility of exergame training in this particular setting, an extension of the training units in an outpatient or home setting was not desired. It should also be mentioned that due to the Covid pandemic, very few patients opted for an inpatient stay, especially at the beginning of recruitment due to the ban on external visitors. This may have resulted in fewer people with Parkinson being included who would have been eligible for the study. In order to recruit as many participants as possible, every person with Parkinson who was in the clinic at this time was informed about the study and checked for suitability. The therapy was not only possible in mild Parkinson patients but also in moderate and advanced ones. As all measurements and trainings were conducted and supervised by the same two investigators on-site, blinding could only be facilitated for the pre-measurement, but not for the post-measurement. However, group assignment could also be blinded as this was conducted by a third person off-site. The exercise intensity in this study may not have been optimal for every patient, given the large interindividual differences in clinical presentation. Quantitative recording of the subjective feeling of difficulty when playing the game would be one way to individually adapt the training load and thus better utilize the capacity of the exergame training.

Another point that should be discussed is the choice and protocol of some outcome measures. The gait analysis only assessed speed, but not gait pattern and stride time variability. However, as patients with PD often show a pathological gait [65], a look at the stride and step length and the sequence of movements would have been helpful for more in-depth results. In previous meta-analyzes from Dockx et al., only changes in the stride and step length were found, but no significant difference in walking speed [66]. However, due to time constraints, this additional measurement had to be dispensed within this study. This also applies to the dual task test, where no special attention was paid to cognitive performance activity. The use of a simple sit-to-stand test such as the modified 30-s seat-to-stand test (m30STS) [67] could also provide additional information about functional mobility. In addition, adherence and adverse events were only considered for exergame-specific training during rehabilitation in this study. In future studies, adherence and adverse events should be considered for all training both in the intervention group and in the control group in order to better understand whether the high adherence of the intervention group depends solely on exergame training or is also related to the rehabilitation stay [50].

In addition, several confounders limit the results of the study. During the intervention period, medication adjustments were made regularly, as some patients were admitted to rehabilitation specifically for this purpose. However, we did not observe any significant differences in the Levodopa equivalent daily dose in both groups at pre- or post-measurements. In addition, not all measurements and training could be carried out when the patient was in an “on”-state. For larger RCTs, it would be important to always carry out the measurements whenever the device is switched on, e.g., to schedule the measurement 2 h after taking the medication. Interestingly, the physiotherapy guideline for Parkinson's disease [68] proposes to perform the TUG in the “off” state in order to increase the accuracy in identifying persons with PD at risk of falling. Since this cognitive–motor exergaming device targets specific fall risk factors, this could provide even better information about its effectiveness. As the primary outcome of this study was feasibility, these confounders were not of great importance, but they should be considered in future effectiveness studies.

## Conclusion and outlook

In summary, this pilot study showed that exergame-based training with the Dividat Senso is a feasible, safe and effective training intervention that can be easily integrated into the therapy plan of people with PD during inpatient rehabilitation. The high adherence rate, the low attrition rate and the high level of acceptance indicate that exergaming has potential to make a rehabilitation program more fun and to increase patient motivation. In this way, the training routine can be maintained and the greatest possible benefit can be derived from the inpatient stay. Another advantage of exergame training is that the therapy can be individualized and adapted very easily and the Dividat Senso itself can also adapt to the performance of the participant in real-time. This is particularly beneficial for people with PD, as there are large individual differences in clinical appearance. Since there is already a positive tendency to improve physical and cognitive functions in this short period of time, it is to be expected that significantly positive effects can be achieved by extending the intervention time. Consequently, future studies should look at extending the period of intervention in the area of outpatient rehabilitation or also as home training. Emphasis should also be placed on adapting the training load to the level of the individual participants in order to adapt the training to the current needs of people with PD. Further attention should be given to the dose–response effects over the course of a rehabilitation program in order to develop more efficient physical training approaches [69].

## Supplementary Information

**Additional file 1:** **Figure.** Weekly Training Plan

## Data Availability

The datasets generated and/or analyzed during the current study are not publicly available due the data protection policy of the Zihlschlacht Rehabilitation Clinic but are available from the corresponding author on reasonable request.

## Abbreviations

PD: Parkinson’s disease
DBS: Deep brain stimulation
RCT: Randomized controlled trial
TUG: Timed-up and go
MSA: Multiple system atrophy
PSP: Progressive supranuclear palsy
MMSE: Mini mental state examination
SUS: System usability scale
NASA-TLX: Nasa task load index
RTT: Reaction time test
TMT: Trail making test
D-KEFS: Delis–Kaplan executive function system
SPPB: Short physical performance battery
5xStS: 5 times sit-to-stand test
ANOVA: Analysis of variance
BMI: Body mass index
MDS-UPDRS: Movement disorder society unified Parkinson’s Disease Rating scale
SD: Standard deviation
IQR: Interquartile range
BBS: Berg balance scale
M30STS: 30 second seat-to-stand test
