# Supplementary figures and images for: Feasibility and effects of cognitive–motor exergames on fall risk factors in typical and atypical Parkinson’s inpatients: a randomized controlled pilot study
Source: Eur J Med Res. 2023 Jan 16;28:30. doi: 10.1186/s40001-022-00963-x (PMC9841664; doi:10.1186/s40001-022-00963-x)

# Additional file

**Additional file 1:** Figure: Weekly Training Plan


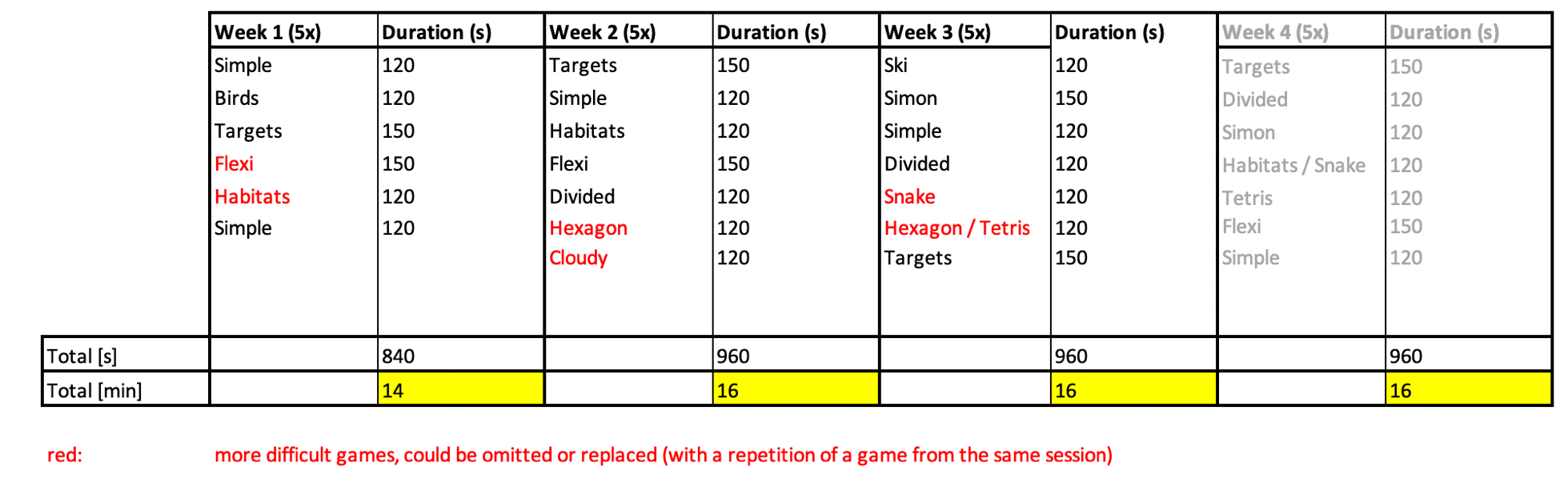

Supplement: Supplementary file 1 — Additional file 1: Figure. Weekly Training Plan [file 40001_2022_963_MOESM1_ESM.docx]
